# Metabolic shift towards oxidative phosphorylation reduces cell-density-induced cancer-stem-cell-like characteristics in prostate cancer *in vitro*

**DOI:** 10.1242/bio.059615

**Published:** 2023-04-06

**Authors:** Hung Wei Lai, Moe Kasai, Shinkuro Yamamoto, Hideo Fukuhara, Takashi Karashima, Atsuhi Kurabayashi, Mutsuo Furihata, Kazuhiro Hanazaki, Keiji Inoue, Shun-ichiro Ogura

**Affiliations:** ^1^ Center for Photodynamic Medicine, Kochi Medical School, Kochi University, Nankoku, 783-8505 Kochi, Japan; ^2^ School of Life Science and Technology, Tokyo Institute of Technology, Yokohama, 226-8501 Kanagawa, Japan; ^3^ Department of Urology, Kochi Medical School, Kochi University, Nankoku, 783-8505 Kochi, Japan; ^4^ Department of Pathology, Kochi Medical School, Kochi University, Nankoku, 783-8505 Kochi, Japan

**Keywords:** Cancer stem cell, Oxidative phosphorylation, Warburg effect, Glycolysis, Prostate cancer

## Abstract

Numerous cancer patients undergoing conventional cancer therapies such as radiotherapy, chemotherapy and surgical tumour removal face relapses several years or even decades later. This may be due to the presence of cancer stem cells (CSCs) that survived said therapies. In this study, we aimed to uncover the relationship between cell density and CSCs, and the role of the Warburg effect in regulating CSC-like characteristics. A prostate cancer cell line, PC3, was used in this study. To investigate the Warburg effect effect and CSC-like characteristics in prostate cancer, we measured the expression levels of glycolysis and OXPHOS-related genes, and performed spheroid forming, cell viability and various glycolysis and OXPHOS-assays. We observed that increased cell density caused a metabolic shift from glycolysis to OXPHOS and higher CSC-like characteristics. However, the use of dichloroacetate (DCA), an inhibitor of the Warburg effect, significantly inhibited the cell-density-induced metabolic shift and CSC-like characteristics. Changes in cell density strongly influenced the preferred metabolic pathway of prostate cancer cells, regulating their CSC-like characteristics. It is possible that DCA, an inhibitor of the Warburg effect, could be a novel drug used to treat CSCs by distinguishing Warburg effect, preventing future cancer relapses.

## Teaser

**Summary:** Cell density affects preferred metabolic pathway of prostate cancer cells, leading to CSC-like characteristics.

## INTRODUCTION

According to the World Health Organization (WHO), about 10 million people worldwide died from cancer in the year 2020, resulting in an estimated total economic cost of $1.16 trillion (WHO, 2021). Cancer has been the leading cause of death in Japan since 1981, comprising 27% of all deaths in 2018 (Tsugane, 2021). Despite the establishment of conventional cancer therapies such as radiotherapy, chemotherapy and surgical tumour removal, many cancer patients often face relapse post-treatment years or even decades later (Aguirre-Ghiso, 2007; Chaffer and Weinberg, 2011). The main reason is believed to be the presence of drug resistant cancer stem cells (CSCs), which survived the therapies (Chaffer and Weinberg, 2011; Kyle et al., 2012). CSCs are found within tumours and characterized by their ability to differentiate and self-renew, entering cell proliferation arrest and reproducing the parent tumour when transplanted into a host (Atashzar et al., 2020). These characteristics of CSCs significantly reduce the effectiveness of conventional cancer therapies as the therapies are commonly targeted at actively proliferating cells (Kusumbe and Bapat, 2009; Talukdar et al., 2019).

Tumours are known to have a highly dense, three-dimensional structure, whereby cancer cells within the tumour exist in a complex microenvironment where they constantly interact with one another (Lin and Chang, 2008; Whiteside, 2008). Cancer cells are known to exhibit various malignancy-related traits when cultured at different cell densities, such as increased proliferative and metastatic capabilities, enhanced lipid metabolism and increased resistance toward cancer drugs (Kuwano et al., 2004; Jayatilaka et al., 2018; Wu et al., 2020; Nakayama et al., 2021; Lai et al., 2021). Nakayama et al. (2016) suggested that an increase in cell density leads to cancer cell dormancy and cell proliferation arrest, both important characteristics of CSCs (Nakayama et al., 2016; Atashzar et al., 2020). Unlike normal cells, cancer cells tend to favour the energy inefficient glycolytic pathway over the more effective oxidative phosphorylation process (OXPHOS) (Warburg, 1956). This preference of cancer cells towards the glycolytic metabolic pathway is widely known as the Warburg effect (Warburg, 1956; Vander Heiden et al., 2009). The inhibition of the glycolytic energy metabolism might have inhibitory effects on cancer cells and CSCs (Pacini and Borziani, 2014; Yuen et al., 2016).

Accordingly, in this study, we attempted to establish the relationship between cell density and CSCs, and its effect on CSCs' energy metabolic pathway preferences, to promote discovery of new treatments against cancer and prevent relapse.

## RESULTS

### Cancer stem cell-like characteristics at various cell densities

Cell density has been reported to induce changes to phenotypic characteristics of cancer cells such as proliferative capability, cancer dormancy and susceptibility to cancer treatment, all characteristics of CSCs (Nakayama et al., 2016; Atashzar et al., 2020). In this study, we wanted to evaluate CSC-like characteristics of PC3 cells at various cell densities by studying four key aspects of a typical CSCs, namely proliferative capability, CSC markers, drug resistance and anchorage-independent proliferation.

**Fig. 1.**
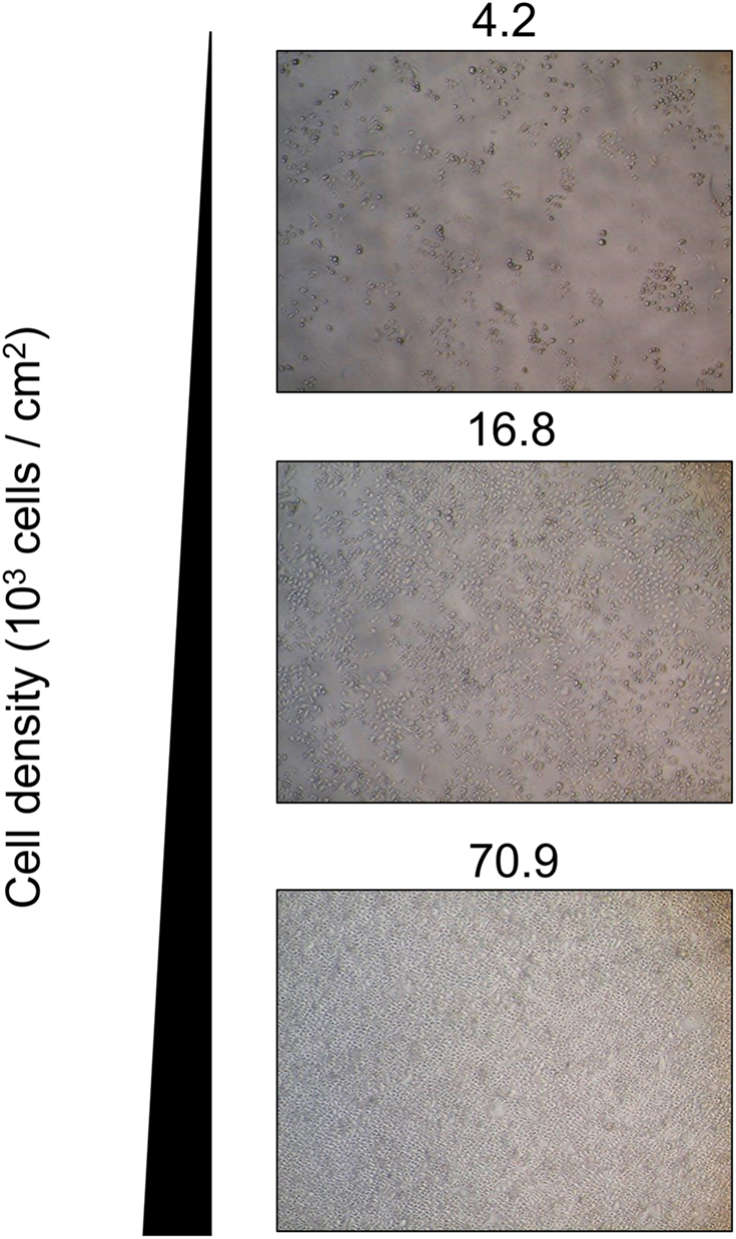
**Microscopy images showing the morphological changes in PC3 cells when cultured at different cell densities.** Scale bars: 500 uM.

**Fig. 2.**
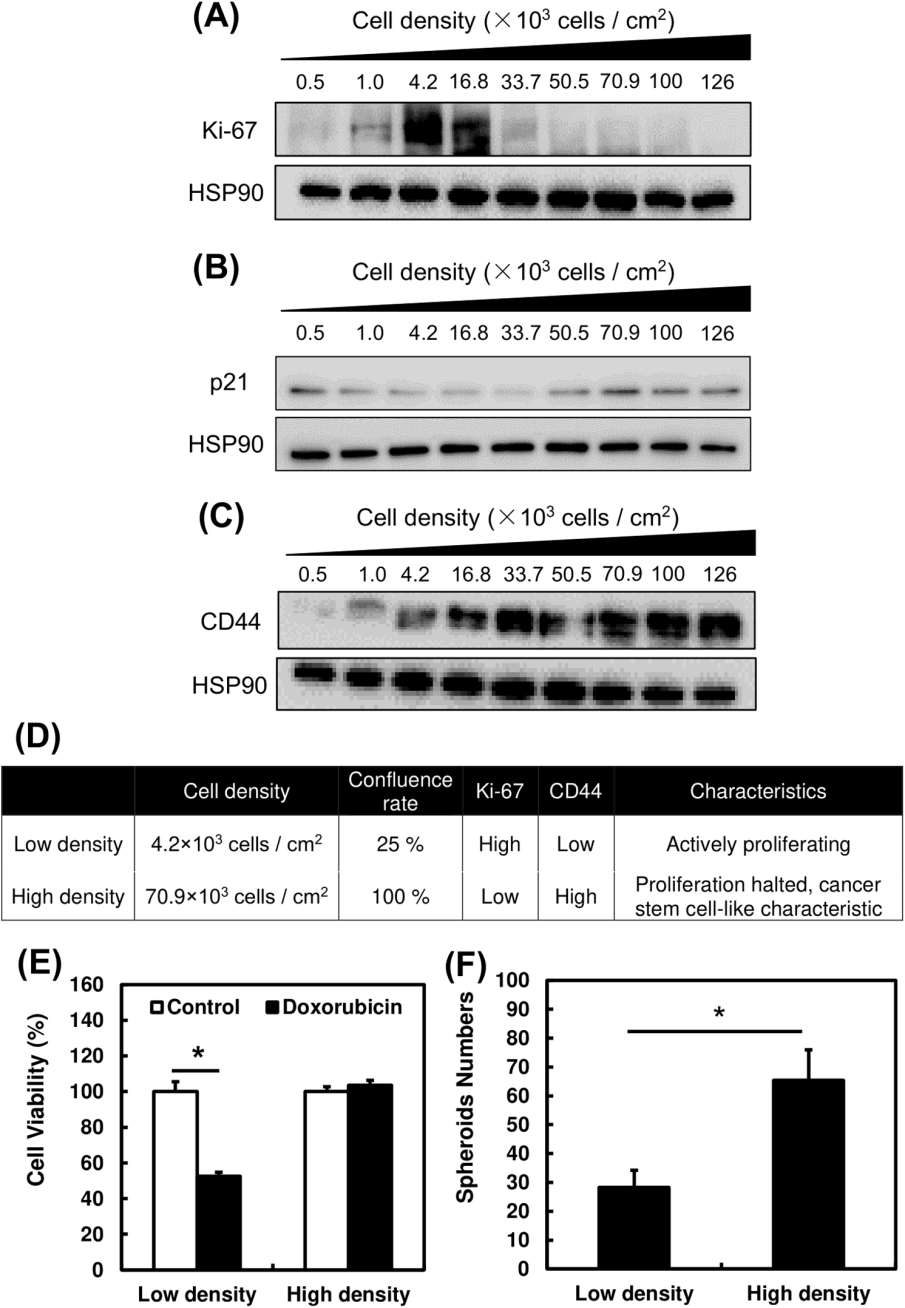
**Evaluation of cancer stem-like properties in PC3 cells at different cell densities.** (A,B) Changes in protein expression levels of proliferation and cancer-stem-cell markers of cells incubated at different cell densities. (C) Table evaluating the confluence rate and characteristics of PC3 cells when incubated at high (70.9×10^3^ cells/cm^2^) and low (4.2×10^3^ cells/cm^2^) cell densities. (D) Cell viability of PC3 cells treated with doxorubicin at high and low cell density respectively. (E) Number of spheroids of PC3 cells grown in ultra-low adhesion dishes at high and low cell densities. A one-way ANOVA (Tukey's test) was performed for each data set to identify differences in mean values between treated and untreated samples; *P<0.05. *n*=3. Bars represent standard deviation (s.d.). Blots are cropped to ease visualization. Unprocessed original blot scans are shown in Fig. S1.

We acquired phase-contrast microscopic images of PC3 cells at different cell densities (Fig. 1). With an increasing number of cells, cell density increases. Next, the proliferative capability of PC3 at different cell densities was evaluated using Ki-67, a proliferative marker (Fig. 2A). Cells at 4.2×10^3^ cells/cm^2^ showed the highest Ki-67 expression level, gradually decreasing with increasing cell density. This suggests a significantly lower proliferative capability of cancer cells when grown at high cell density. The expression of proliferative marker, Ki-67 was confirmed by evaluating expression of cell arrest marker, p21 (Fig. 2B). Expression of p21 is low at cell densities which Ki-67 exhibited high expression and vice versa.

CD44 was used as a CSC marker (Fig. 2C). It is a transmembrane glycoprotein found on the cell surface that binds to the extracellular matrix and activates EGFR and ErbB-2. These activations promote cell migration and differentiation (Atashzar et al., 2020). Fig. 2C show that the expression level of CD44 gradually increases with cell density. This suggest that the CSC-like characteristics of PC3 cells increase with increasing cell density.

Based on Ki-67, p21 and CD44 expression levels, we determined that cells cultured at 70.9×10^3^ cells/cm^2^ (high density) most closely represents CSC-like cells while cells at 4.2×10^3^ cells/cm^2^ (low density) represent actively proliferating cells (Fig. 2D). The effect of doxorubicin, a conventional drug for treating cancer, on cells cultured at high and low density was evaluated; no significant changes were observed in cell viability in high density samples (Fig. 2E). On the other hand, low density samples showed a 50% decrease in live cells. This suggests that cells with CSC-like characteristics are more resistant to cancer drugs.

CSCs have been reported to possess anchored-independent proliferative capability (Bahmad et al., 2018). Therefore, we used an ultra-low adherent dish to grow spheroids and study this characteristic. The number of spheroids with diameter >500 μm were counted after 7 days of incubation. The number of spheroids formed is >2 times higher in high-cell-density samples than in low-cell-density samples (Fig. 2F). This suggests significantly higher anchored-independent proliferative capability of cells cultured at high density. Figs 1 and 2 suggest that PC3 cells at high density had more CSC-like characteristics such as higher proliferative ability and higher drug resistance.

### Changes in glycolytic metabolism in PC3 cells under different cell density

**Fig. 3.**
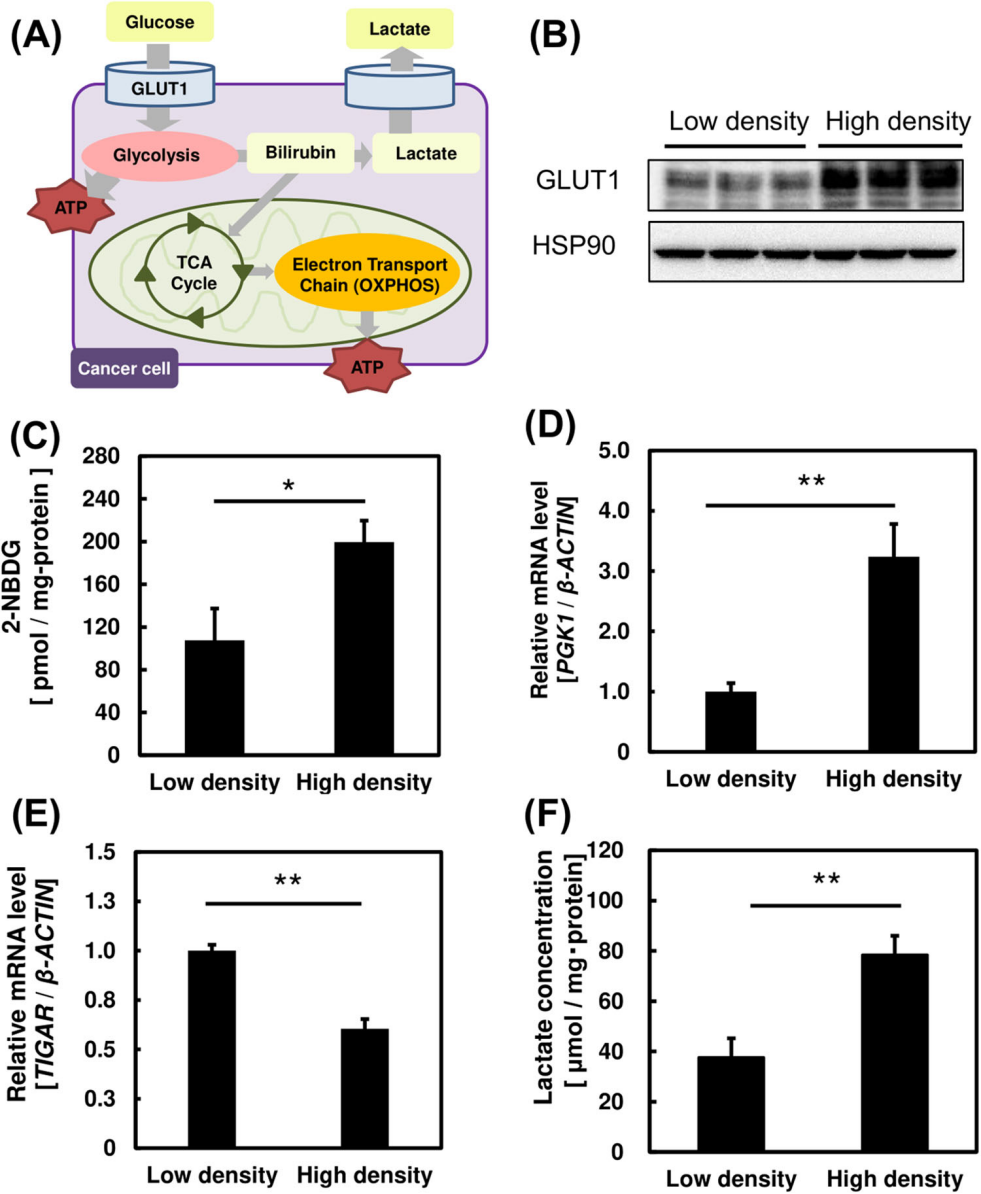
**Changes in glycolytic metabolism in PC3 cells at high and low cell density.** (A) Schematic illustration showing the energy metabolism in cancer cells. (B) Protein expression level of glucose transporter 1 (GLUT1) when incubated at high and low cell density tested by western blotting. (C-E) Bar graphs showing 2-NBDG concentration in PC3 cells grown at high and low cell densities (C); mRNA expression levels of the glycolysis-related gene, *PGK1* (D)*,* and glycolysis inhibitory gene, *TIGAR,* at high and low cell densities (E). (F) Bar graph showing lactate production of PC3 cells grown at high and low cell densities. A one-way ANOVA (Tukey's test) was used for each data set to calculate differences in mean values between treated and untreated samples; *P<0.05; **P<0.01. *n*=3. Bars represent standard deviation (s.d.). Blots are cropped to ease visualization. Unprocessed original scans of blots are shown in Fig. S2.

Fig. 3A shows two energy metabolic pathways in a cell, the glycolytic pathway and oxidative phosphorylation (OXPHOS) in the electron transport chain. In this section, we evaluated changes in the glycolytic metabolism in PC3 cells at different cell densities by analyzing the protein expression level of glucose transporter 1 (GLUT1), changes in glucose uptake, glycolysis-related genes and lactate production. High-cell-density samples were prepared at 70.9×10^3^ cells/cm^2^ and low-cell-density samples at 4.2×10^3^ cells/cm^2^. GLUT1 expression was significantly higher in cells grown at high density (Fig. 3B). The uptake of the fluorescence-tagged glucose analogue (2-NBDG) was significantly higher in high-density samples (Fig. 3C). These findings in that the increase in glucose uptake is possibly related to that in GLUT1 expression.

Next, the glycolytic metabolism was evaluated by studying changes in expression levels of genes involved in glycolysis, such as the phosphoglycerate kinase 1 (*PGK1*) and TP53-induced glycolysis regulatory phosphatase (*TIGAR*) (Fig. 3D and E). *PGK1* is the first enzyme involved in ATP generation in the glycolytic pathway by catalyzing the conversion of 1,3-diphosphoglycerate to 3-phosphoglycerate (Hu et al., 2017). On the other hand, high expression of *TIGAR* inhibits the production of fructose-2,6-bisphosphate, preventing allosteric inhibition of glycolysis-related enzymes, namely phosphofructokinase 1 and fructose 1,6-biphosphatase. This would suppress glycolysis, decreasing the production of reactive oxygen species (ROS) (Bensaad et al., 2006). In Fig. 3D and E, we observed increased *PGK1* and decreased TIGAR expression levels at high density, indicating a higher glycolytic metabolism in PC3 cells incubated at high density. Finally, we measured the lactic acid, a glycolytic end-product, in the medium at different cell densities (Rogatzki et al., 2015). Fig. 3F shows a higher lactic acid production in cells grown at high density, suggesting a higher glycolytic metabolism in PC3 cells grown under those conditions.

These results suggest that the glycolytic metabolism is enhanced and more preferred in cancer cells at high cell density, possibly through the increased expression level of a glucose transporter, glycolysis-related genes, and the higher generation of a glycolytic end-product. Next, we studied changes on the OXPHOS pathway at different cell densities.

### Effect of cell density on oxidative phosphorylation in PC3 cells

**Fig. 4.**
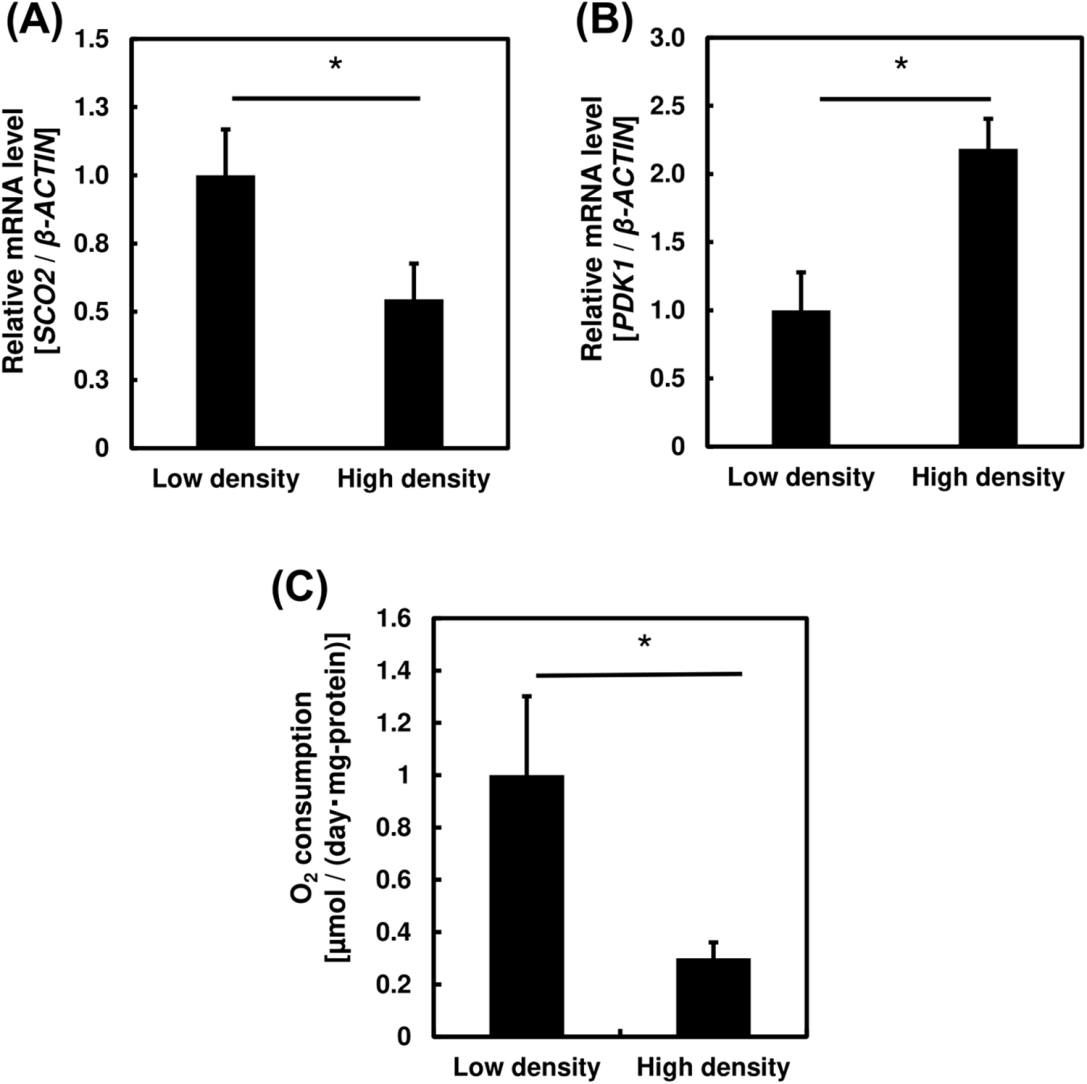
**Bar graphs showing effect of cell density on oxidative phosphorylation in PC3 cells.** (A) mRNA expression levels of the oxidative phosphorylation-related gene, *SCO2*, in cells incubated at high and low cell densities. (B) mRNA expression levels of the oxidative phosphorylation inhibitory gene, *PDK1*, in cells when incubated at high and low cell density. (C) Oxygen consumption of PC3 cells when incubated at high and low cell density. One-way ANOVA (Tukey's test) was used to calculate differences in mean values between treated and untreated samples; *P<0.05; **P<0.01. *n*=3. Bars represent standard deviation (s.d.).

Previous findings showed enhanced glycolytic metabolism of PC3 cells under high cell density. In this section, we attempted to clarify how cell density affects OXPHOS in prostate cancer cells by examining the mRNA expression levels of two OXPHOS-related genes, synthesis of cytochrome c oxygenase 2 (*SCO2*) and pyruvate dehydrogenase kinase 1 (*PDK1*). *SCO2* is a gene involved in promoting OXPHOS in the mitochondria while *PDK1* suppresses pyruvate dehydrogenase complex, preventing OXPHOS and pyruvate oxidation in the mitochondria (Wanka et al., 2012; Atas et al., 2020). Fig. 4A and B showed lower *SCO2* and increased *PDK1* expression levels, suggesting a lower OXPHOS activity in PC3 cells cultured at high cell density.

The oxygen consumption rate (OCR) of PC3 cells was significantly lower in high density cultures (Fig. 4C). This suggest lower OXPHOS activity in said conditions. Based on the results on Fig. 3, we hypothesized that cancer cells exhibit an enhanced glycolytic metabolism and lower OXPHOS when cultured at high density, in the so-called Warburg effect (Warburg, 1956). Next, we examined the effect of the addition of a Warburg inhibitor to PC3 cells on these energy metabolic pathways.

### Effect on glycolytic metabolism in PC3 cells following dichloroacetate addition

**Fig. 5.**
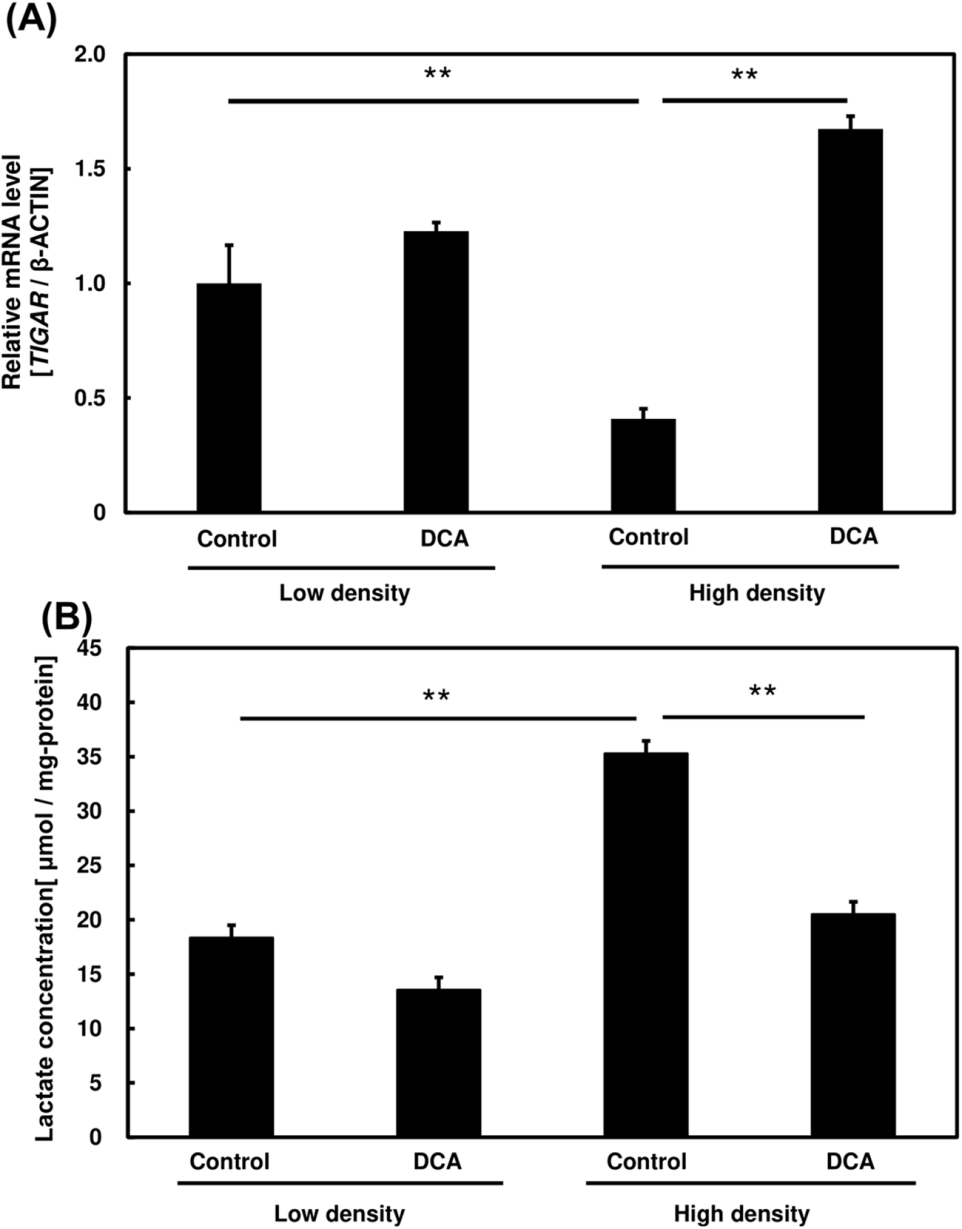
**Effect of dichloroacetate (DCA) addition on glycolytic pathways in PC3 cells at different cell densities.** (A) mRNA expression levels of the glycolysis inhibitory gene, *TIGAR*, in cells incubated at high and low density in the presence or absence of DCA. (B) Lactate production of PC3 cells treated with or without DCA. One-way ANOVA (Tukey's test) was used to calculate differences in mean values between treated and untreated samples; **P<0.01. *n*=3. Bars represent standard deviation (s.d.).

We investigated the effects of dichloroacetate (DCA), an inhibitor of the Warburg effect, on the cell-density-induced Warburg effect in PC3 cells (Liu et al., 2019). PC3 cells grown at high and low densities were subjected to 40 mM DCA treatment before measuring the changes in glycolysis-inhibitory gene, *TIGAR* and lactic acid production (Fig. 5).

In agreement with Fig. 3E, *TIGAR* expression was significantly lower in high density samples (Fig. 5A) before the addition of DCA (*P*<0.01). This suggests glycolytic activity suppression following DCA administration. This lactate concentration was evaluated in Fig. 5B. DCA administration showed a significant reduction of lactate levels in both samples, suggesting lower glycolytic production due to inhibited glycolysis. These findings suggest DCA can distinguished Warburg effect through suppression of glycolytic activity.

### Effect on oxidative phosphorylation in PC3 cells after dichloroacetate addition

**Fig. 6.**
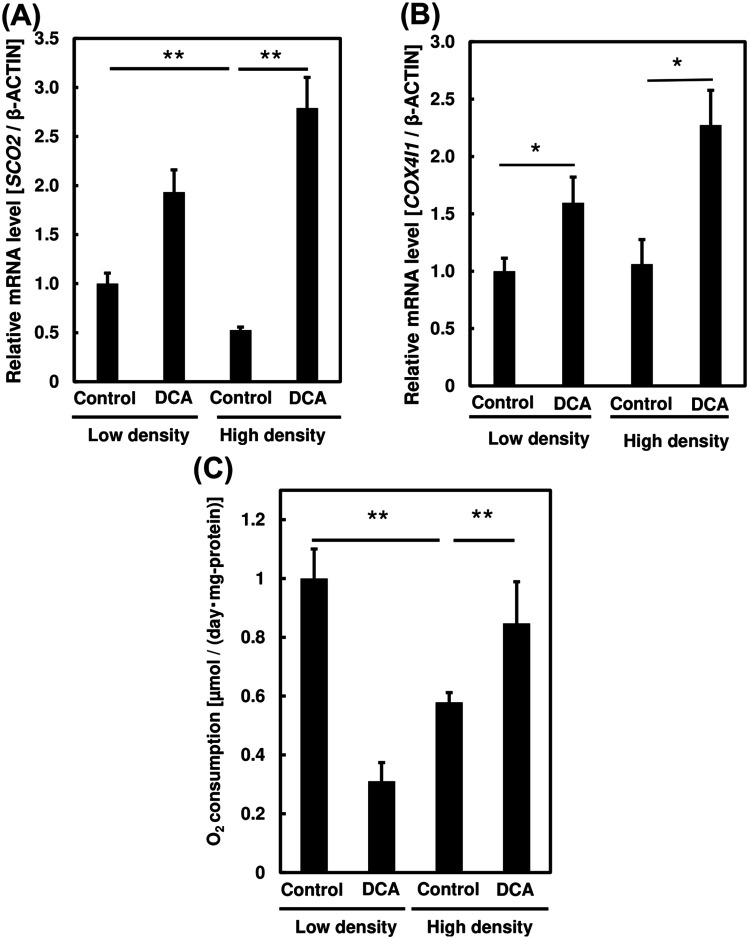
**Effect of dichloroacetate (DCA) addition on oxidative phosphorylation in PC3 cells.** mRNA expression levels of the oxidative phosphorylation-related genes, (A) *SCO2*, and (B) *COX4I1*, following DCA addition in cells incubated at high and low density. (C) Oxygen consumption of PC3 cells following DCA addition in cells incubated at high and low density. One-way ANOVA (Tukey's test) was used to calculate differences in mean values between treated and untreated samples; *P<0.05; **P<0.01. *n*=3. Bars represent standard deviation (s.d.).

We then investigated the effect of DCA on OXPHOS activity in PC3 cells after DCA addition to high and low cell density cultures. After DCA (40 mM) was applied to PC3 cells mRNA expression levels of OXPHOS-related genes, namely *SCO2* and *COX4*, and OCR were measured (Fig. 6). The expression levels of both *SCO2* and *COX4* increased after DCA addition in both high and low cell density cultures (Fig. 6A and B). This suggests that DCA reduces glycolytic activity and increases OXPHOS in PC3 cells, both typical of the hallmark of Warburg effect (Warburg, 1956).

Fig. 6C shows increased OCR following DCA addition. This increase in O_2_ consumption is believed to be due to higher OXPHOS activity in PC3 cells. DCA administration increases OCR in PC3 cells, indicating a shift in energy metabolic pathways from glycolysis to OXPHOS. These findings suggest that DCA plays a role in identifying a cell density-induced Warburg effect in PC3 cells.

### Changes in cancer-stem-cell-like characteristics in PC3 cells following dichloroacetate addition at different cell densities

We attempted to clarify the effect of DCA addition on the CSC-like characteristics in PC3 cells by evaluating the expression level of the CSC marker, CD44, following DCA addition at different cell densities.

**Fig. 7.**
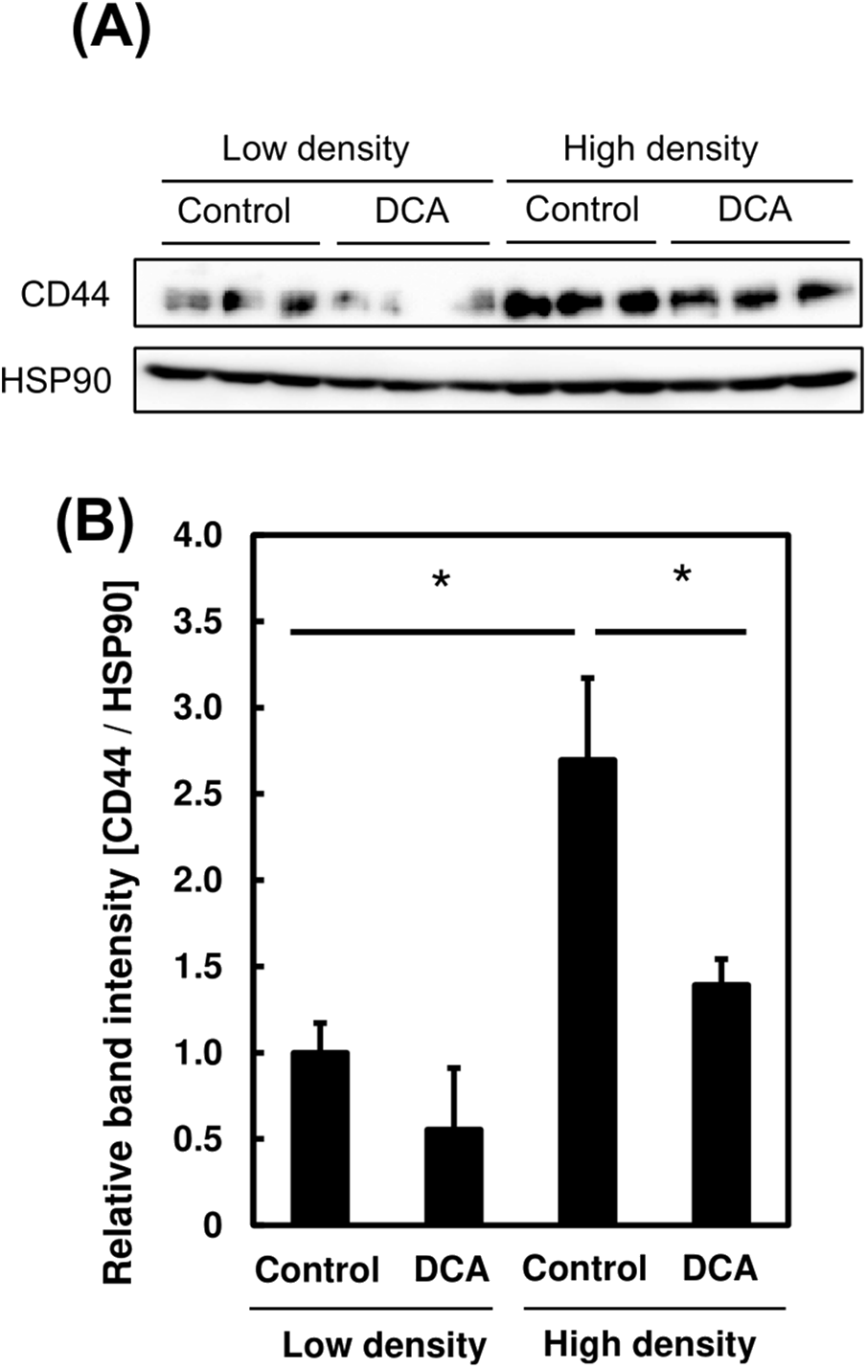
**Changes in energy metabolism in PC3 cells after dichloroacetate (DCA) addition at high and low cell density.** (A) Lactate production of PC3 cells grown at high and low density. (B) Oxygen consumption of PC3 cells at high and low cell density. (C,D) Changes in protein expression level of cancer stem cell markers in the presence or absence of DCA at high and low cell density. One-way ANOVA (Tukey's test) was used to calculate differences in mean values between treated and untreated samples; *P<0.05. *n*=3. Bars represent standard deviation (s.d.). Blots are cropped to ease visualization. Unprocessed original scans of blots are shown in Fig. S3.

DCA was added to the medium to a final concentration of 40 mM and cultured for 24 h following incubation of PC-3 cells at different cell densities for 72 h. CD44 protein expression level was measured using western blotting. Similar to the results in Fig. 6C, CD44 expression level was significantly higher in PC3 cells incubated at high density (Fig. 7A). We observed a significant decrease in CD44 protein expression when treated with DCA at high cell density. The decreased CD44 expression level at low cell density was not significant whereas CD44 expression is halved when treated with DCA at high cell density (*P*<0.05; Fig. 7B). This suggests that the CSC-like characteristics induced under high-density culture conditions are inhibited by DCA. In summary, CSC-like characteristics are more prevalent in prostate cancer cells at high cell density, when induced by an enhanced Warburg effect.

## DISCUSSION

The Warburg effect, the tendency of cancer cells to favour the inefficient glycolytic pathway rather than OXPHOS to generate energy despite being in an environment with abundant oxygen supply, has been an interesting topic that has intrigued scientists since its first discovery (Warburg, 1956). Although a decade has passed since its first discovery, clinical trials on the Warburg effect have shown mixed results due to the complexity of the cancerous microenvironment, prompting further understanding of its fundamentals (DeBerardinis and Chandel, 2020).

Several reports have evaluated the relationship between the Warburg effect and CSCs, suggesting that a metabolic shift from OXPHOS to glycolysis contributes to high cancer stemness (Yuen et al., 2016; Liu et al., 2021). CSCs are known to have higher preference for glycolysis over OXPHOS as a source of energy metabolism, a symbolic characteristic of the Warburg effect. Therefore, the removal of Warburg effect might affect the energy acquirement of CSCs for their metabolic processes (Zhu et al., 2020). However, this is the first study to suggest a relationship between cell density and CSC-like characteristics, and its possible connections with cancer dormancy (Nakayama et al., 2016). The four most notable methods of identifying CSC characteristics are evaluation of proliferative capabilities, resistance toward cancer drugs, CSC markers and anchored-independent proliferation (Bahmad et al., 2018; Atashzar et al., 2020). We observed significantly reduced proliferative capabilities of PC3 cells, as indicated by Ki-67 expression levels, beyond a cell density of 16.8×10^3^ cells/cm^3^ (Fig. 2A). This indicated a lower cell proliferative capability, a characteristic of CSC. This finding was supported by the correlation of expression of p21 with Ki-67 from Fig. 2B. Fig. 2E also showed increased drug resistance of CSC-like cells following administration of doxorubicin, a commonly used cancer drug. Using CD44 as a CSC marker has clinical relevance, although further clinical research is required in the future (Kalantari et al., 2017). We found that prostate cancer cells exhibited more CSC-like characteristics when grown at high cell density (Fig. 1). These characteristics are also the main factors that make CSCs difficult to treat using conventional cancer chemotherapy, since these drugs target actively replicating cancer cells (Kusumbe and Bapat, 2009; Talukdar et al., 2019). Therefore, a novel drug that could target CSCs would be highly helpful for scientists and medical doctors in cancer treatment.

In this study, we found increased glycolytic activity under high cell density by the evaluation of *PGK1* and *TIGAR* expression levels, two important genes involved in glycolysis (Fig. 3). *PGK1* plays an important role in the progression of cancer through its involvement in the regulation of cancer cell proliferation and tumorigenesis, while *TIGAR* inhibits glycolysis and protect cells from ROS-associated apoptosis (Bahmad et al., 2018; Liu et al., 2019; Atashzar et al., 2020). These results are further supported by the increased lactic acid production from the glycolytic metabolism and decreased OXPHOS activity at high cell density (Figs 3 and 4). These findings suggest an enhanced Warburg effect under high cell density in prostate cancer cells. We believe that high cell density increases the frequency of cell contact and cell–cell communication, leading to an enhanced Warburg effect.

DCA is an inhibitor for the Warburg effect that carries out its action through the inhibition of aerobic glycolysis and promotion of pyruvate oxidation, particularly through the inhibition of activity of PDK1 by activating pyruvate dehydrogenase, a rate-limiting enzyme of aerobic glycose oxidation (Khan et al., 2017). Several papers have suggested that DCA possesses therapeutical relevance in the treatment of CSCs and may contribute to eradication of CSC population respectively (Tataranni et al., 2019; Tataranni and Piccoli, 2019). This drug has succeeded in suppressing tumour growth in preliminary studies and is now undergoing several clinical trials as new potential cancer drug. DCA potentially inhibits cancer growth if aerobic glycolysis is active in targeted cancer cells (Wanka et al., 2012). Fig. 5 shows that cell-density-induced elevated glycolysis was inhibited following DCA administration. These results are further strengthened by recovery in OXPHOS activity and OCR following DCA addition particularly in high density samples (Fig. 6). This suggests the cell-density-induced metabolic shift in PC3 cells closely resembled the Warburg effect in cancer.

Based on Fig. 7, we found that distinguished Warburg effect following DCA administration led to a significant reduction in expression level of CD44 in PC3 cells. The reduction in CD44 expression levels suggest lower CSC-like characteristics; distinguishing the Warburg effect could potentially reduce the CSC population. These findings suggest that DCA may be used as a drug to treat CSCs through suppression of Warburg effect.

In summary, we found that CSC-like characteristics are influenced by prostate cancer cell density. CSC-like characteristics may be dependent on the energy metabolic pathway of cancer cells. In addition, DCA could be a novel drug for CSCs by specifically targeting the Warburg effect. In short, our results suggest that a metabolic shift from the glycolytic pathway to OXPHOS may reduce cell-density-induced CSC-like characteristics in prostate cancer.

## MATERIALS AND METHODS

### Cells and cell culture

The human prostate-cancer cell line, PC3, was obtained from the American Type Culture Collection (ATCC). The cells were cultured in RPMI 1640 culture medium containing 10% Fetal bovine serum (FBS) and 10% ABAM, at 37°C in a 5% CO_2_ incubator. Experiments were performed at a cell density of 50–80% confluence.

### Biochemicals

RPMI-1640 culture medium and antibiotic–antimycotic mixed stock medium (ABAM) were purchased from Nacalai Tesque (Kyoto, Japan). FBS was purchased from Equitech-Bi (Kerrville, TX, USA) and dicholoroacetate (DCA) from Sigma-Aldrich Corporation (Tokyo, Japan).

### Western blotting analyses

Western blotting analyses were carried out as previously described (Lai et al., 2019). We used polyclonal anti-human Ki-67 antibody (Abcam, Cambridge, UK; 1:1000), anti-human p21 antibody (Invitrogen, Waltham, MA, USA, 1:500), anti-human CD44 antibody (Abcam, Cambridge, UK; 1:200), anti-human GLUT1 antibody (Abcam, Cambridge, MA, USA; 1:500), anti-human GAT2 antibody (Medical & Biological Laboratories, Nagoya, Japan; 1:1000), and anti-human HSP90 antibody (Santa Cruz Biotechnology, TX, USA; 1:200 dilution) as primary antibodies. Secondary antibodies were horseradish peroxidase (HRP)-conjugated anti-mouse (Cell Signaling Technology, Beverly, MA, USA) and anti-rabbit IgG (Santa Cruz Biotechnology, Dallas, TX, USA) concentrates, diluted 3000 times in tris-buffered saline (TBST) solution.

### Cell viability test

After seeding, the cells were cultured in an incubator at 37°C and 5% CO_2_ for 72 h and inoculated at a cell density of 0.2×10^6^ cells/cm^2^. After culturing overnight, the medium were replaced and 5 µM doxorubicin (Wako, Osaka) was added, and 24 h later, the number of viable cells was measured by Trypan Blue staining. Trypan Blue solution (Nacalai Tesque, Kyoto) was dissolved in 0.5% (w/v) using phosphate buffered saline (PBS). Cells and supernatant were collected and mixed in a 1:1 ratio of Trypan Blue aqueous solution. Ten microliters of solution were dropped onto a haemocytometer and inserted into Countess® IIFL (ThermoFisher Scientific, USA) for quantification of living and dead cells. Cells appearing white without staining were identified as living cells and blue-stained cells were identified as dead. The number of living cells without doxorubicin was set to 100% under each cell density, and cell viability was calculated.

### Spheroid forming assay

Cells were seeded and cultured in an incubator at 37°C, 5% CO_2_ for 72 h. The cell density was then adjusted to 500 cells/cm^2^, and the cells were inoculated into an ultra-low adhesion dish (Corning, NY, USA) containing Tumorsphere Medium XF (Takara Bio, Shiga) as medium. The number of spheroids with diameter >500 µm after incubation at 37°C and 5% CO_2_ for 7 days was recorded.

### Measurement of glucose uptake ability

The amount of glucose uptake by cell was determined by measuring the concentration of fluorescence-tagged glucose, 2-deoxy-2-[(7-nitro-2,1,3-benzoxadiazol-4-yl)]-D-glucose (2-NBDG), in the cell. 2-NBDG was purchased from Cayman Chemical, MI, USA. Cells were seeded and cultured for 72 h in an incubator at 37°C and 5% CO_2_. Next, the medium was replaced with a glucose-free medium and 2-NBDG was added to achieve a final concentration of 100 μM. The cells were then cultured for 25 min. To extract the 2-NBDG accumulated in the cells, the cells were washed with PBS, and 300 μl of 0.1 M NaOH was added to each well to prepare a cytolytic solution. Part (250 μl) of the solution was collected in a 1.5 ml tube, and the protein was denatured with the same amount of 1.0 M perchloric acid-methanol (1:1, v/v) to isolate intracellular 2-NBDG. Centrifugation was performed at 4°C and 10,000×***g*** for 10 min to remove the denatured protein, and the collected supernatant was analyzed with a fluorescence spectrophotometer (RF-5300PC, Shimadzu Corporation, Kyoto). An excitation wavelength of 465 nm and a detection wavelength of 550 nm were used to measure 2-NBDG's fluorescence intensity. In addition, the protein concentration of the solution was measured using the Bradford method.

### Real-time quantitative reverse transcription-polymerase chain reaction (qRT-PCR)

Cells were seeded and cultured in an incubator at 37°C and 5% CO_2_ for 72 h, medium was then replaced and cells were further cultured for another 24 h under different conditions. Total RNA was extracted using NucleoSpin® RNA II (Macherey-Nagel, Mannheim, Germany) and underwent reverse transcription to cDNA using the PrimeScript RT reagent Kit with gDNA Eraser (TaKaRa, Shiga, Japan). A two-step quantitative reverse transcription–polymerase chain reaction (qRT–PCR) was performed using Thermal Cycler Dice® Real Time System Single and mRNA transcripts were quantified using SYBR Premix Ex Taq II (TaKaRa, Shiga, Japan). The primers used in this study are shown in Table 1. An initial heat denaturation step was performed at 95°C for 30 s, followed by a run of 45 cycles for 5 s at 95°C and 60°C for 1 min. The dissociation curve was analyzed immediately after amplification to confirm the PCR product specificity. The data were analyzed using Thermal Cycler Dice analysis software (TaKaRa, Shiga, Japan). The Ct value was calculated using the crossing point method, and relative mRNA expression levels were calculated from the calibration curve. The mRNA expression level of each sample was standardized by that of actin. All procedures were performed according to manufacturer's instructions.

**Table 1.**
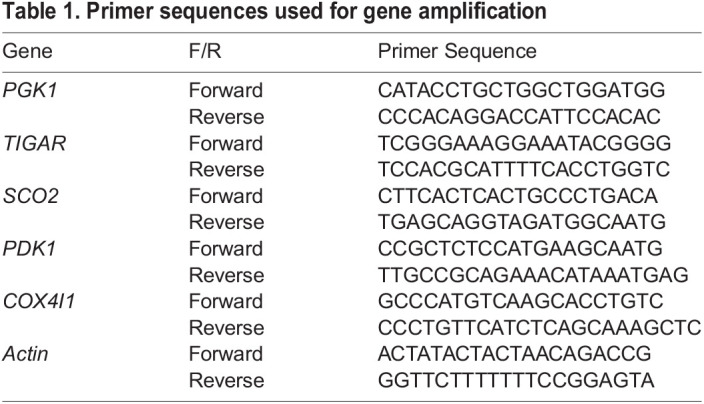
Primer sequences used for gene amplification

### Lactic acid production analyses

The amount of lactic acid produced was measured by colorimetric analysis using Glycolysis Cell-Based Assay Kit (Sigma-Aldrich). Cells were seeded and cultured for 72 h in an incubator at 37°C and 5% CO_2_. Then, the medium was replaced by a serum-free medium, and cells were cultured under each condition for 24 h. The supernatant was collected in a 2 ml tube, centrifuged at 1500 rpm for 5 min to remove dead cells, and 10 μl of the culture supernatant was placed into each well of a 96-well plate. A glycolysis assay L-lactate standard, cell-based assay buffer, glycolysis assay substrate, glycolysis assay enzyme mixture, and glycolysis assay cofactor were placed in wells and allowed to react at room temperature for 30 min. The absorbance of the reaction solution was measured at 450 nm using a Multiskan FC absorbance microplate reader (ThermoFisher Scientific, San Jose, CA, USA), and the value was then calculated from the calibration curve.

### Oxygen consumption in culture media

The amount of dissolved oxygen in the medium was measured using a 24-channel SDR Sensor Dish® Reader (Presens, Regensburg, Germany). Cells were seeded in 24-well OxoDish® (Presens) and cultured for 72 h in an incubator at 37°C and 5% CO_2_. Thereafter, the culture medium was replaced with fresh medium and incubated for another 24 h before measuring the amount of dissolved oxygen. The oxygen concentration of the medium before and after the 24-h culture was measured, and the value obtained by subtracting the measured value after the culture from the measured value before the culture taken as the oxygen consumption; the value was corrected by the amount of protein in the cells. Liquid paraffin (800 μl) was added to prevent oxygen elution in the air during culture.

### Statistical analysis

Microsoft Excel 2010 was used for data analysis in this study. A one-way ANOVA (Tukey's test) was performed for each data set to identify differences in mean values between treated and non-treated samples; at two levels of significance, *P*<0.05 and *P*<0.01.

## Supplementary Material

10.1242/biolopen.059615_sup1 Supplementary information
