# Supplementary material for: Metabolic shift towards oxidative phosphorylation reduces cell-density-induced cancer-stem-cell-like characteristics in prostate cancer in vitro
Source: Biol Open. 2023 Apr 6;12(4):bio059615. doi: 10.1242/bio.059615 (PMC10110405; doi:10.1242/bio.059615)
Supplement: Supplementary information [file biolopen-12-059615-s1.pdf]

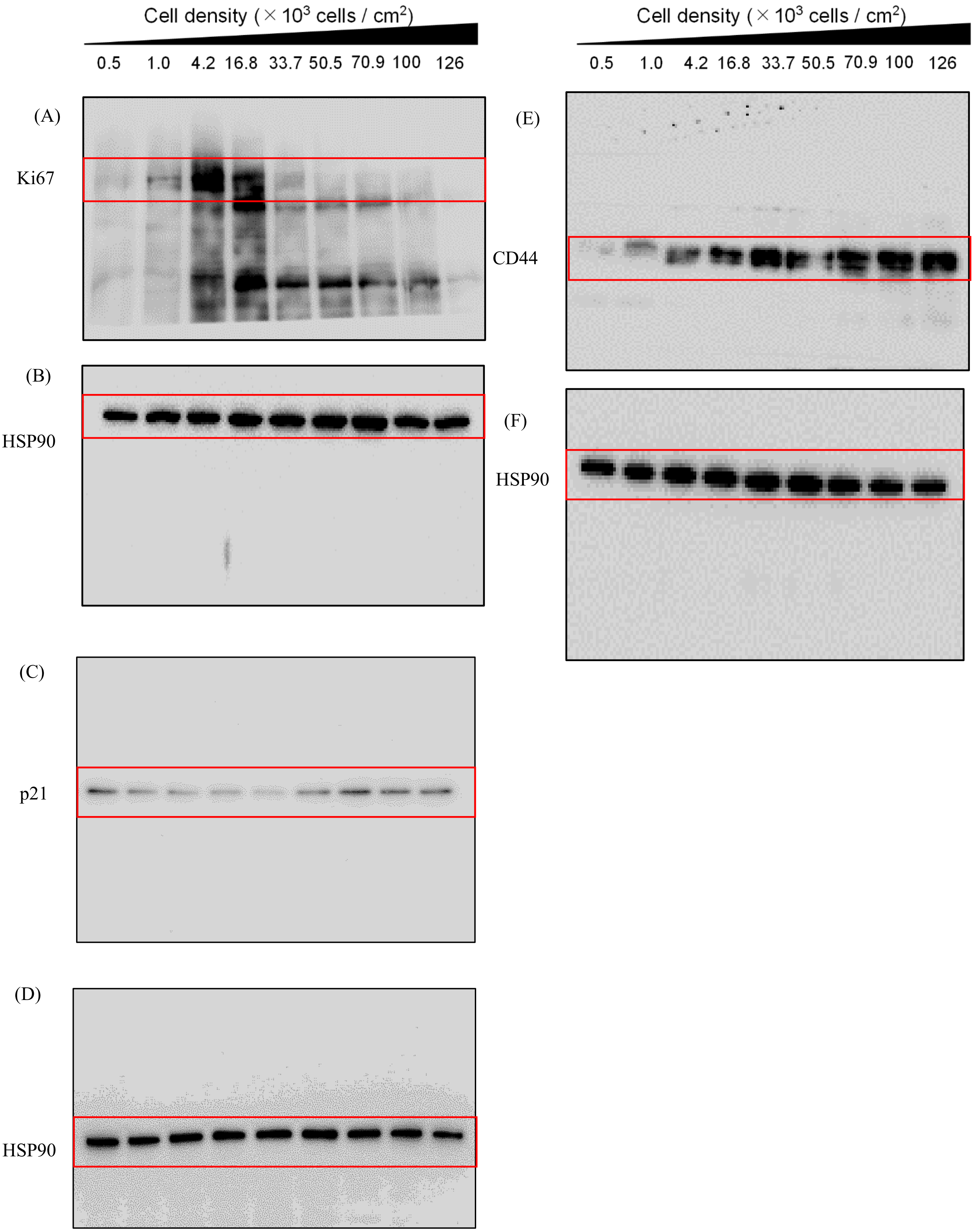

Fig. S1. Original blots showing results from Fig. 2A, 2B & 2C. Protein expression of (A) Ki67, (B) HSP90, (C) p21, (D) HSP90, (E) CD44 and (F) HSP90 in PC3 cells under different cell densities.

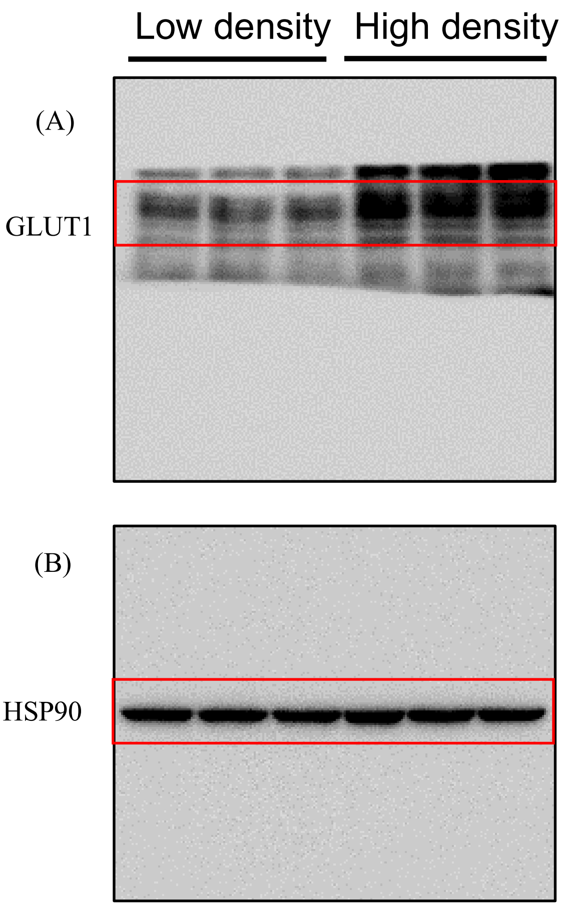

Fig. S2. Original blots showing result from Fig. 3B. Protein expression of (A) GLUT1 and (B) HSP90 in PC3 cells under different cell densities.

Biology Open • Supplementary information

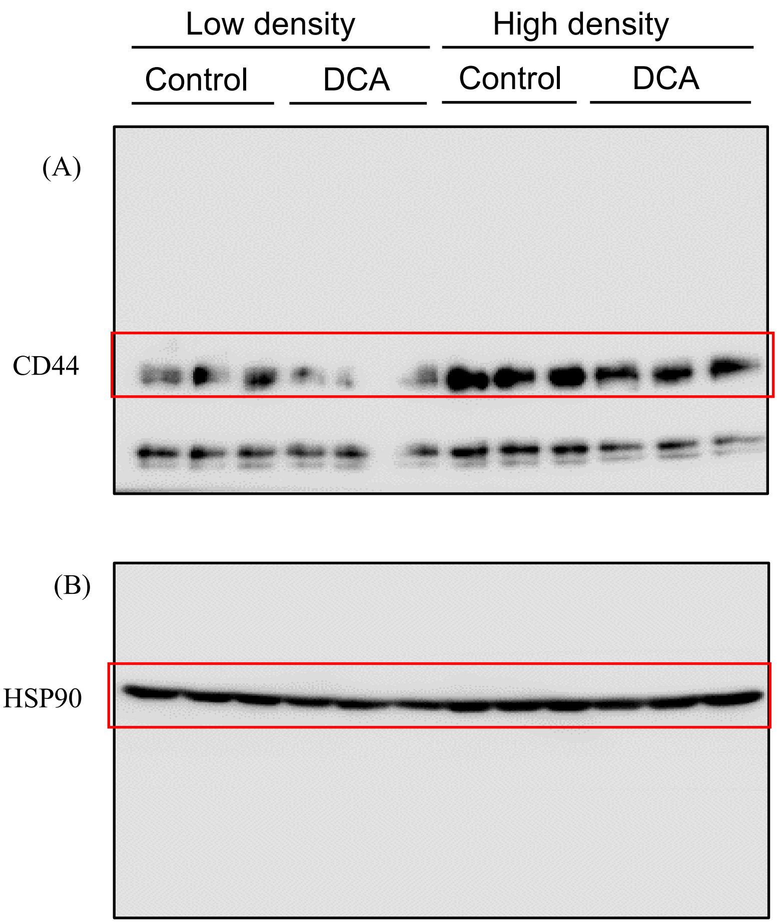

Fig. S3. Original blots showing results from Fig. 7A. Protein expression of (A) CD44 and (B) HSP90 in PC3 cells under different cell densities.
